# Design and Development of an Upper Limb Rehabilitative Robot with Dual Functionality

**DOI:** 10.3390/mi12080870

**Published:** 2021-07-24

**Authors:** Md Rasedul Islam, Md Assad-Uz-Zaman, Brahim Brahmi, Yassine Bouteraa, Inga Wang, Mohammad Habibur Rahman

**Affiliations:** 1 Richard J. Resch School of Engineering, University of Wisconsin-Green Bay, Green Bay, WI 54311, USA; 2 Mechanical/Biomedical Engineering Department, University of Wisconsin-Milwaukee, Milwaukee, WI 53211, USA; assaduz2@uwm.edu (M.A.-U.-Z.); rahmanmh@uwm.edu (M.H.R.); 3 Electrical and Computer Engineering, Miami University, Oxford, OH 45056, USA; brahmib@miamioh.edu; 4 Department of Computer Engineering, College of Computer Engineering and Sciences, Prince Sattam Bin Abdulaziz University, Al-Kharj 11942, Saudi Arabia; yassine.bouteraa@isbs.usf.tn; 5 Control and Energy Management Laboratory (CEM Lab), Ecole Nationale d Ingenieurs de Sfax (ENIS), Institut Superieur de Biotechnologie de Sfax (ISBS), University of Sfax, Sfax 3038, Tunisia; 6 College of Health Sciences, University of Wisconsin-Milwaukee, Milwaukee, WI 53211, USA; wang52@uwm.edu

**Keywords:** rehabilitation, upper limb, dual functionality, exoskeleton, end effector, upper arm forces, sensorized cuff

## Abstract

The design of an upper limb rehabilitation robot for post-stroke patients is considered a benchmark problem regarding improving functionality and ensuring better human–robot interaction (HRI). Existing upper limb robots perform either joint-based exercises (exoskeleton-type functionality) or end-point exercises (end-effector-type functionality). Patients may need both kinds of exercises, depending on the type, level, and degree of impairments. This work focused on designing and developing a seven-degrees-of-freedom (DoFs) upper-limb rehabilitation exoskeleton called ‘u-Rob’ that functions as both exoskeleton and end-effector types device. Furthermore, HRI can be improved by monitoring the interaction forces between the robot and the wearer. Existing upper limb robots lack the ability to monitor interaction forces during passive rehabilitation exercises; measuring upper arm forces is also absent in the existing devices. This research work aimed to develop an innovative sensorized upper arm cuff to measure the wearer’s interaction forces in the upper arm. A PID control technique was implemented for both joint-based and end-point exercises. The experimental results validated both types of functionality of the developed robot.

## 1. Introduction

With the increase of hemiplegic stroke patients day by day [1], upper limb rehabilitation to regain lost mobility seems to be required more than ever nowadays. It was well established from past research that the success of a rehabilitation program heavily depends on its repetitiveness and intensity. The repetitive nature, tediousness, precision, explicit feedback, and intensity of therapy are some key factors that make robot-aided rehabilitation popular for upper limb impairments. For instance, robotic devices can provide therapy for an extended period, irrespective of skills and fatigue, compared to manual therapy [2]. They can also work in multiple degrees of freedom with virtual reality interfaces and provide therapy ranging from passive to active rehabilitation. This leverage over traditional therapy could increase the efficiency and effectiveness of therapists by alleviating the labor-intensive aspects of physical rehabilitation of post-stroke patients [3]. To provide rehabilitation therapy to individuals with upper limb impairment, several research prototypes have been developed to date [4,5,6,7,8,9,10,11,12,13,14,15,16,17,18]. Among these prototypes, very few were transferred into a commercial product, as they have limitations (in both hardware and control design), which are discussed in following paragraphs.

Based on the correspondence of the robot’s joints onto the wearer, existing rehabilitative robotic devices can be grouped into two main categories, i.e., exoskeleton-type devices [19,20,21,22] and end-effector-type devices [7,23,24]. Exoskeleton-type devices can map the motion and torque to the corresponding human joint, making them have better guidance and control over individual joints. It is possible to mimic the whole arm motion with such devices if they require seven DoFs for general upper limb motions. On the other hand, end-effector-type devices cannot map onto the corresponding human joints; hence, they cannot produce whole arm motion. However, unlike their counterparts, end-effector-type devices are suitable for end-point exercises [25] and it is easier to accommodate spastic patients with a rotated shoulder, flexed elbow, pronated forearm, flexed wrist, and so on [26,27,28]. Note that upper limb spasticity is observed in approximately one-third of the stroke survivors [29,30]. Seemingly, as an individual role, the exoskeleton-type and end-effector-type robots have apparent limitations in serving a wide variety of patients with different degrees of upper limb impairments and providing various types of therapeutic exercises. It is worth mentioning that existing robotic devices are either exoskeleton type, such as [22,30,31,32], or end-effector type [4,7,10,18]. Including the functionalities of both exoskeleton-type and end-effector-type devices in a single robot would serve a wide variety of patients, depending on the type, level, and degree of impairments. Moreover, this dual functionality would facilitate the rehabilitation of patients with spasticity. Inspired by the idea of dual functionality, a seven-DoF upper limb exoskeleton robot called ‘u-Rob’ was engineered in this research to function as both an exoskeleton-type and end-effector-type robot.

Better human–robot interaction (HRI) is always desired in the design of upper limb exoskeletons. For instance, the forces and torques generated in exoskeleton joints must be successfully transferred to human joints. Proper alignment between human joints and exoskeleton joints would make sure that this transfer occurs. The primary source of misalignment can stem from the exoskeleton’s shoulder joint if its center of rotation (CR) remains fixed during a maneuver. Most of the existing exoskeletons were developed with a fixed-CR shoulder joint as a three-DoF ball-and-socket joint [8,17,33,34,35,36,37,38]. However, some exoskeletons were developed with this kinematic structure in mind [14,39,40,41]. These adjustments come with the tradeoff of a reduced ROM and a complex design. To address the mobility of the shoulder joint’s CR for increased ROM, two parallel mechanisms that were designed in the authors’ previous research were adapted in u-Rob’s kinematic structure. These mechanisms were used to create an ergonomic shoulder skeleton in u-Rob.

In addition to the alignment, monitoring the interactive forces between the wearer and exoskeleton robot is a must to ensure safety, comfort, and better HRI. However, existing devices ignore interaction forces during passive rehabilitation. Such an oversight can become costly if the wearer has a stiff arm. Furthermore, current rehab devices have ignored measuring interaction forces at the wearer’s upper arm [6,41,42]. To measure the interaction forces at the upper arm, a sensorized upper arm cuff was designed and developed for u-Rob’s upper arm module.

Despite the development of numerous prototypes, research on robotic devices for upper limb rehabilitation is still a growing field and demands novel approaches to solve key limitations in hardware design (e.g., human–robot interface and dual functionality). In this research, an upper limb exoskeleton robot was designed to address some key limitations found in existing exoskeletons. The main contributions of this research are as follows:

(1) A novel seven-DoF upper limb exoskeleton robot called ‘u-Rob’ was developed that functions as both an exoskeleton-type robot and an end-effector-type robot to perform joint-based and end-point exercises, respectively.
(2) A sensorized upper arm cuff was designed and incorporated into u-Rob to measure and monitor the interaction forces at the upper arm.

The remainder of the work is structured as follows. Section 2 presents a detailed description of the developed u-Rob exoskeleton. The kinematic modeling, dynamic modeling and control of the exoskeleton system are presented in Section 3. Section 4 presents the experimental results and discussion. Finally, the paper ends with the conclusion presented in Section 6.

## 2. Development of u-Rob

### 2.1. General Design Requirement

A rehabilitative robot’s design requirements largely depend on the range of motion and limb segment to be included [43,44,45]. The complex joint articulation of the human upper limb makes the design of exoskeleton robots difficult. The human upper limb is mainly composed of seven degrees of freedom (DOFs) to provide seven general motions at the shoulder (i.e., abduction–adduction, vertical flexion–extension), upper arm (i.e., internal–external rotation), elbow (i.e., flexion–extension), forearm (i.e., pronation–supination), and wrist (i.e., radial–ulnar deviation, flexion–extension). In addition, at the shoulder joint, there are two passive movements in the frontal (i.e., elevation–depression) and sagittal planes (protraction–retraction). u-Rob has seven DoFs and three modules, namely, a three-DoF shoulder module to support motion at the shoulder and upper arm, a two-DoF elbow module to support the motion at the elbow and forearm, and a two-DoF wrist module to support wrist motions.

The suitable ranges of motions for the proposed exoskeleton robot were chosen based on the existing literature [46,47,48,49,50]. Table 1 shows the selected ranges of motion of u-Rob. A large workspace allowed for designing a rehabilitation protocol with a variety of exercises.

### 2.2. Development Procedure

To develop the proposed u-Rob exoskeleton robot, the following steps were carried out.

The very first step in the development of u-Rob’s hardware was to study the anatomy and biomechanics of the human upper limb to find the safe ranges of motion [47,50,51].

Anthropometric parameters (e.g., arm length, arm segment’s weight, and segment inertia) of the upper limbs were studied to obtain u-Rob’s link parameters. To choose the suitable link parameters for u-Rob, the length, weight, inertia, and center of gravity location of the upper arm, forearm, and wrist were studied for typical white, Asian, African-American, Hispanic, and Latino adult men and women [50,51,52]. These parameters were also used in the simulation to choose actuators [52]. Note that u-Rob is wearable for adult men and women with heights ranging from 4 foot 7 inches to 6 foot 2 inches.

**Figure 1:**
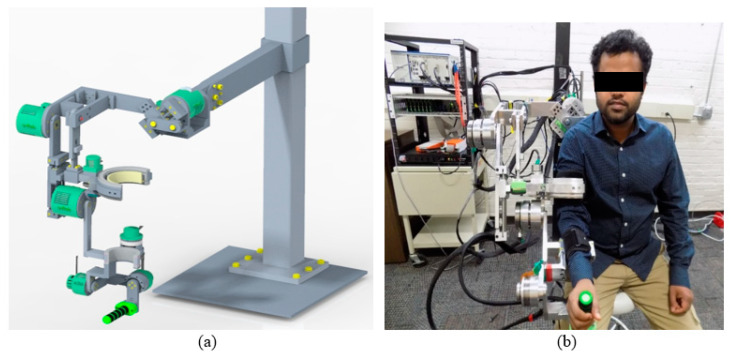
(**a**) Rendered CAD model of the u-Rob exoskeleton robot and (**b**) a subject wearing the u-Rob exoskeleton robot.

With the selected ranges of motion and lengths of the various segments, the mechanical components were designed, and a complete CAD model of the proposed exoskeleton robot (shown in Figure 1a) was developed in PTC Creo (version 5.0, Needham, MA, USA). This model provided the center of gravity and inertia properties of the proposed exoskeleton robot’s segments.

The CAM of the mechanical components was designed in Fusion 360 (version 2.0.6258, Autodesk Inc., San Rafael, CA, USA).

CNC milling, centering, and drilling operations were used to fabricate u-Rob’s components.

u-Rob was made ready to function (please see Figure 1b) with all the components fabricated and assembled with the required screws and fasteners.

Throughout the following sub-sections, details of u-Rob’s design and development are presented. Furthermore, design specifications and selected components of the developed u-Rob are presented in Table 2.

### 2.3. Shoulder Module

**Figure 2:**
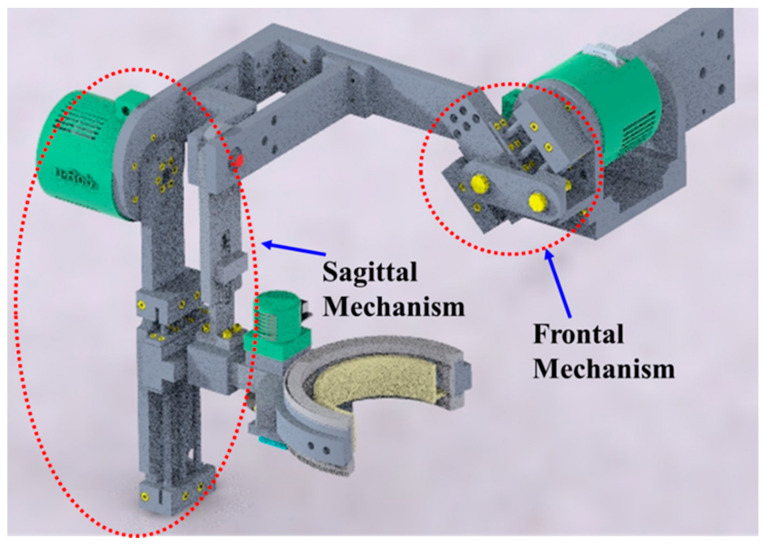
Ergonomic shoulder motion support part of the proposed exoskeleton robot.

**Figure 3:**
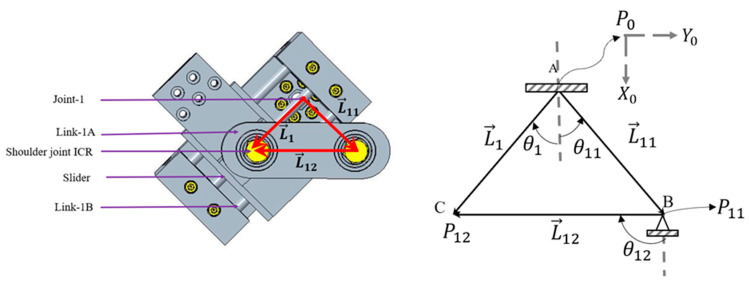
CAD diagram (**left**) and vector formation (**right**) of the frontal mechanism.

**Figure 4:**
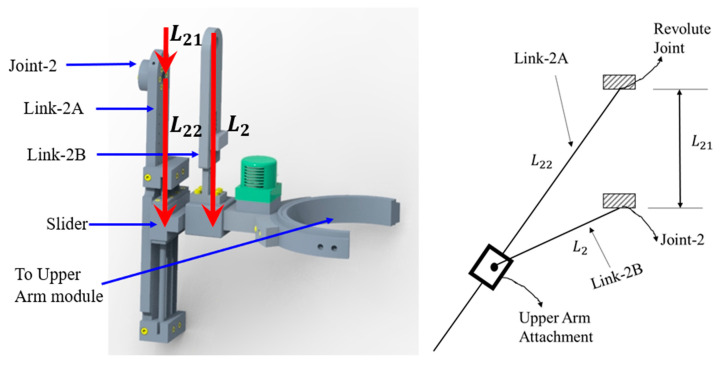
CAD diagram (**left**) and vector formation (**right**) of the sagittal mechanism.

According to human upper limb anatomy, there are three general motions (i.e., shoulder abduction–adduction in the frontal plane, shoulder vertical or horizontal flexion–extension in the sagittal plane, and internal–external rotation in the transverse plane) in the shoulder. These three movements are also known as glenohumeral (GH) articulations. The intersecting point of the axes of these three motions is often known as the center of the GH joint (also known as the shoulder joint’s instantaneous center of rotation (ICR)). In addition to these three general motions, there are two other motions (i.e., elevation-depression and protraction–retraction) in the frontal plane and sagittal plane of the human body that produce shoulder abduction–adduction and flexion–extension, respectively; the conventional ball-and-socket joint cannot provide movement to the shoulder joint’s center of rotation. To realize additional movements in the frontal and sagittal planes, the shoulder motion support part of u-Rob was designed using a hybrid approach by incorporating both parallel and serial mechanisms, as shown in Figure 2. Two parallel mechanisms, namely, the frontal and sagittal mechanisms, as shown in Figure 3 and Figure 4, were used in the design of the ergonomic shoulder module. These mechanisms were described in detail in the author’s previous research [53,54,55,56]. When combined, these mechanisms allow for the mobility of the shoulder joint’s instantaneous center of rotation by providing movement in the frontal and sagittal planes, respectively. Altogether, there were three actuated (active) DoFs and two passively actuated DoFs used in the ergonomic shoulder module. All the actuated DoFs are revolute joints and are responsible for doing the abduction–adduction (joint-1), vertical flexion–extension (joint-2), and internal–external rotation (joint-3), whereas two passive DoFs are responsible for moving the shoulder joint’s ICR (passive joint-1) during abduction–adduction and doing the protraction–retraction (passive joint-2) during vertical flexion–extension. Note that the intersection of joint-1, joint-2, and joint-3 locates the shoulder joint’s instantaneous center of rotation.

**Figure 5:**
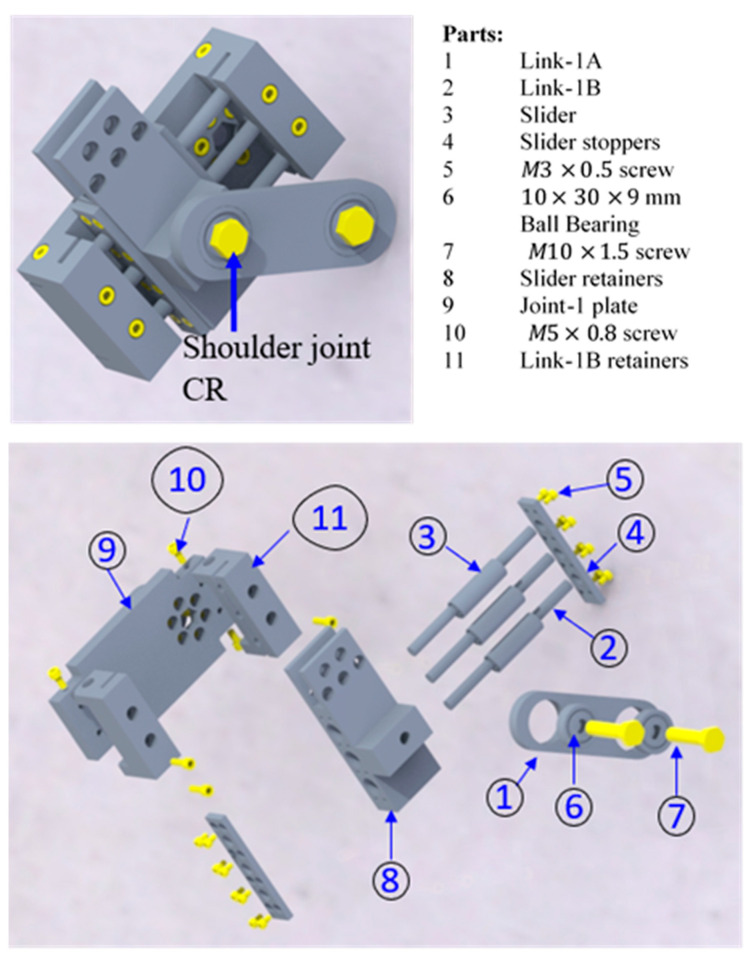
Exploded view of the frontal mechanism.

Figure 5 shows the exploded view of the frontal mechanism with all the parts used in the fabrication. All the parts, except standard elements (e.g., bearings, bushing, stainless steel shaft), were machined out of aluminum 6061. To provide linear motion, three standard (LM8LUU Linear bushing) sliders (part 3) and three 8 mm stainless steel shafts (part 2) were used, as shown in Figure 5. Note that three sets of sliders were used to prevent rotation of the slider around the axis of the shaft. The shafts were made to fit into the slider bore, whereas the sliders were inserted into the bores of the slider retainers (part 8). To prevent horizontal translation of the slider itself, two preregular plates (part 4) were fastened using M4 screws (part 5) at both ends of the slider retainer (part 8). Note that the slider retainer also connects the joint-2 assembly. To hold the shaft, two block parts (part 11) with the appropriate groove and slot were fabricated. These blocks were mounted on the plate (part 9) attached to joint-1. The link-1A (part 1) contains two standard ball bearings (6200Z 10 mm × 30 mm × 9 mm double-sealed ball bearings). These bearings were pressed fit and provide bearing support at two M10 screws. The left end of the link-1A (part 1) connected the shoulder joint CR on part 8 and was hinged at the right end.

**Figure 6:**
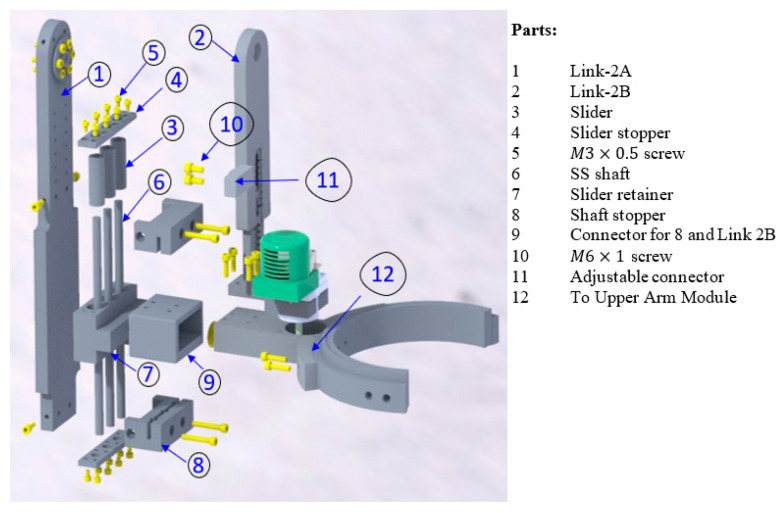
Exploded view of the sagittal mechanism.

Figure 6 shows an exploded view of the sagittal mechanism with the parts used to fabricate it. All the parts, except standard elements (e.g., bearings, bushing, stainless steel shafts), were machined out of aluminum 6061. To provide linear motion along the shaft axis, three standard (LM8LUU linear bushing) sliders (part 3) and three 8 mm stainless steel shafts (part 6) were used, as shown in Figure 6. The purpose of using three sliders was to prevent rotation of the slider retainer about the shaft axis. Link-2A (part 1) houses the shaft retainer (part 8). The sliders (part 3) were inserted into the slider retainer (part 7) that provides the linear motion along the shaft axis. In order to make a connection between link-2B (part 2), the slider retainer (part 7), and the upper arm module, a 3D printed part (part 9) was used. The adjustability of link 2B was achieved using an aluminum machine part (part 11) that was placed at the desired slot.

### 2.4. A New Sensorized Upper Arm Cuff

Designing an upper arm cuff with force sensors always remains a crucial problem. Unlike the wrist sensor, placing sensors in the upper arm is difficult in terms of providing the adequate space that standard three-axis and six-axis force sensors may require. Instead of putting the sensors in the serial link, this research came up with a new approach that places the sensors on the upper arm circular cuff. Both button-type force sensors and flexible pressure sensors can be placed on the cuff wall; however, flexible pressure sensors’ accuracy and resolution are debatable. Therefore, in this research, three button-type force sensors were used.

**Figure 7:**
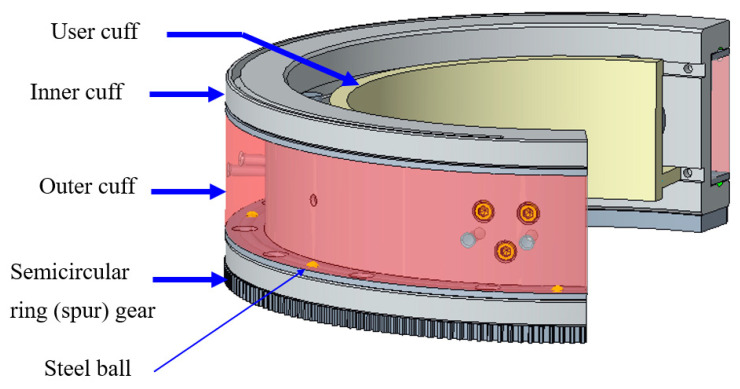
Upper arm sensorized cuff assembly.

Figure 7 shows the sensorized cuff assembly for the upper arm. In order to allow for rotation in the upper arm, as shown in Figure 7, the outer cuff remains stationary while the inner cuff rotates. The reduction of actuator-3’s speed was achieved in two stages. First, the motor speed was reduced using a harmonic reducer (CSF-11-100-2XH-F, Harmonic Drive LLC, US Headquarter, Dunham Ridge, MA, USA). After that, the speed was further reduced using a standard anti-backlash spur gear. Finally, the motion was transmitted to the custom-made semi-circular ring (spur). This gear was fastened to the inner cuff. Thus, the inner cuff produces the rotation to realize upper arm internal–external movement.

**Figure 8:**
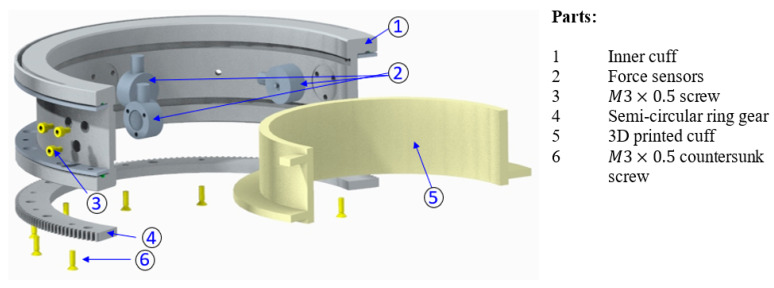
Exploded view of the upper arm sensorized cuff assembly.

To measure the upper arm force, three button-type force sensors (part 2) were mounted on the inner cuff (part 1), as shown in Figure 8. The sensors were fastened using three M3 screws (part 3) that were spaced at 120°. The user cuff (part 5) was placed inside the rectangular groove of the inner cuff (part 1). Two ball plungers mounted on the inner cuff (part 1) maintain the initial tension of the user cuff on the force sensors. To produce the upper arm rotation, the inner cuff houses a custom-made semi-circular spur gear (part 4). This gear meshes with an anti-backlash spur gear (Model LFS-D6-80, Nordex, Inc, Brookfield, CT, USA) that transfers output motion from the joint-3 actuator. The bearing action between the inner cuff and outer cuff (coming from joint-2) is provided by a bearing sleeve. The bearing action in the sleeve was achieved using steel balls, which were placed inside the circular guide. Thus, bearing action is provided during the relative movement of the inner and outer cuff.

The inner cuff was machined in both a lathe and a computer numerical control (CNC) mill; an aluminum 6061 hollow round bar was used in the fabrication. The user cuff was 3D printed; hence, it can be easily made for different user sizes. The semi-circular spur gear was machined out of stainless steel.

### 2.5. Elbow Module

**Figure 9:**
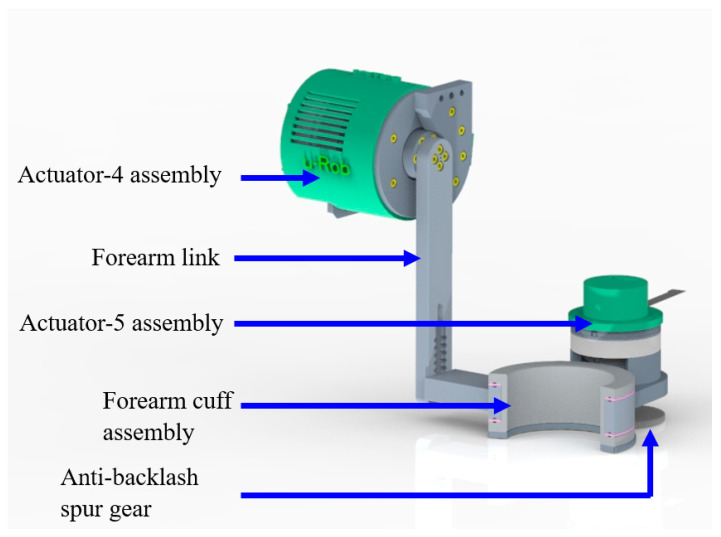
Elbow and forearm motion support part.

**Figure 10:**
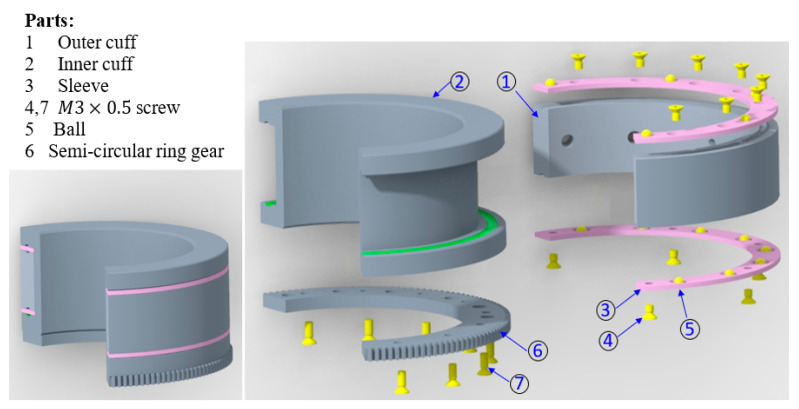
Forearm cuff assembly and its exploded view.

The elbow module is responsible for realizing flexion–extension at the elbow and pronation–supination at the forearm. The elbow flexion–extension is achieved through the actuator-4 assembly, which consists of a motor, a harmonic reducer, and an output adapter. The output of the actuator-4 assembly was fastened to the forearm link, as shown in Figure 9. The forearm link houses the forearm cuff assembly, the exploded view of which is shown in Figure 10. This cuff is similar to the upper arm cuff, therefore a detailed description is avoided here.

In the fabrication of the forearm motion support part, aluminum (aluminum 6061) was used for the forearm link, outer cuff, and inner cuff. Both lathe and CNC milling were used in the fabrication. The machining operations included facing, 2D adaptive clearing, contouring, groove cutting, turning, drilling, and chamfering. The custom-made semi-circular ring (spur) gear was fabricated out of stainless steel (stainless steel 304). The sleeves in the forearm cuff assembly were 3D printed using 1.75 mm PLA filament. The balls used in the forearm cuff assembly are standard 4 mm stainless steel balls.

### 2.6. Wrist Module

**Figure 11:**
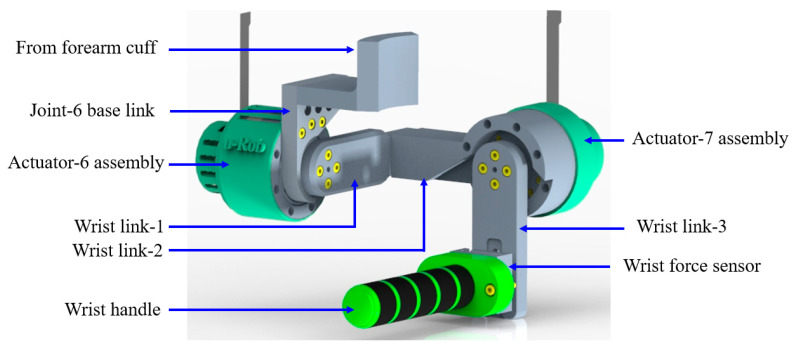
Wrist motion support part of the proposed exoskeleton robot.

**Figure 12:**
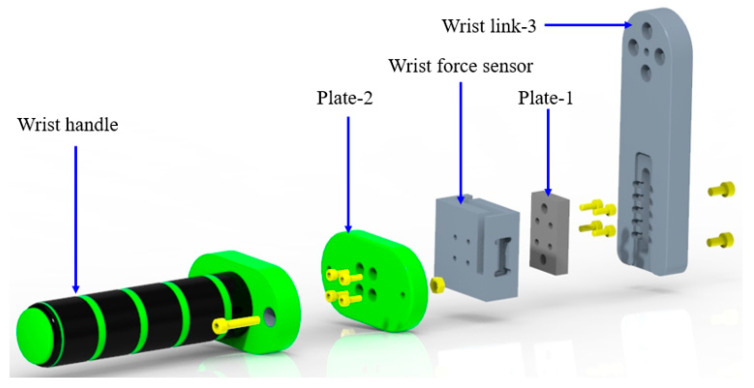
Exploded view of the integration of the wrist force sensor into the proposed exoskeleton robot.

The wrist module of the proposed exoskeleton functionality of u-Rob consists of two revolute joints to provide wrist radial–ulnar deviation and flexion–extension. Moreover, a force sensor was placed at the wrist handle to sense three Cartesian forces exerted by the user. As shown in Figure 11, the actuator assembly for joint-6 was mounted on the joint-6 base link; the base link was rigidly connected to the output of the forearm cuff. The output of actuator-6 was then fastened to wrist link-1. Note that the base link was designed so that it acts as a physical stopper for wrist link-1. The other end of wrist link-1 was rigidly fastened to wrist link-2, which houses the actuator assembly for joint-7. The output of actuator-7 was connected to wrist link-3 with a force sensor in between. The integration of the force sensor into the wrist module is shown in Figure 12.

During the fabrication of the wrist motion support part, aluminum was used for the fabrication of the joint-6 base link, wrist link-1, wrist link-2, wrist link-3, and plate-1. The computer-aided manufacturing (CAM) of these parts was designed in AutoCAD Fusion 360 and machined in CNC. The operations used during the milling included facing, 2D adaptive clearing, contouring, drilling, and chamfering. The wrist handle and plate-2 were 3D printed.

### 2.7. Actuators and Reducers

All the actuators of u-Rob are brushless DC motors. A Maxon EC90 flat 90 W (PN 323772) motor was used in joint-1, 2 and 4. Maxon EC45 flat 70 W (PN 397172) motor was used for joint-3. To actuate joint-5, 6 and 7, a Maxon EC45 flat 70 W (PN 339281) was used.

To reduce the motor speeds, harmonic reducers (strain wave gears) were used. Because of being advantageous over traditional gears, this kind of reducer has been increasingly used over the past several years. The reason for selecting a harmonic reducer in the u-Rob exoskeleton robot was to provide zero-backlash motion. In u-Rob, harmonic reducers from two companies were used. Joint-1, 2, 3, and 4 used harmonic reducers from Harmonic Drive LLC, US Headquarter, Dunham Ridge, MA, USA, whereas joint-5, 6, and 7 used reducers from Leaderdrive, Suzhou, China.

### 2.8. Mass and Inertia Properties of the Proposed Exoskeleton Robot

The mass and mass moment of the inertia about the center of gravity (CG) for the segments of the proposed exoskeleton robot were determined in the CAD environment in PTC Creo and are presented in Table 3. Mass properties were also validated by checking the mass of the real parts of the proposed exoskeleton robot. The segment was determined according to the movement. For instance, the first segment is every element situated after the joint-1 actuator output and before the joint-2 actuator output.

### 2.9. Safety

Safety is paramount as upper limb exoskeleton robots have close interactions with wearers. Human–robot interaction must be designed to ensure safe operations. An HRI should include safety measures in the mechanical, electronic, and control designs for the robot’s safe use. Mechanically, safety is ensured by placing physical stoppers in the robot’s structure to prevent it from going beyond the chosen ROM; safety can also be confirmed by designing links and robot parts in such a way that adjacent links act as physical stoppers in extremes. In u-Rob, adjacent links were designed to work as inherent physical stoppers for the chosen range of motion. Electronically, by setting current and voltage limits in motors, robot joints can be stopped from going beyond the permissible ROM. In control design, saturation can be set for torque, force, velocity, and position to ensure the wearer’s safety if the robot malfunctions. u-Rob’s control algorithm includes thresholds for ROM, velocity, force, and torque; one can easily change and set them from u-Rob’s graphical user interface.

## 3. Kinematics, Jacobian, and Dynamics

### 3.1. Kinematics

The kinematic parameters (position, velocity, and acceleration) of robotic manipulators can be determined using analytical or geometric approaches. The analytical approach involves the vector formation of kinematic parameters and their vector operation, leading to obtaining the kinematic model. However, in the case of a serial manipulator, robotic researchers have extensively been interested in using modified Denavit–Hartenberg parameters [57] due to their simplicity and ease of use in applications (e.g., developing forward kinematics, inverse kinematics, Jacobians, and dynamic model). Since u-Rob is composed of both serial linkage and parallel mechanisms, a combined approach was applied to find the kinematics. The analytical approach was used to find the kinematics of parallel mechanisms and is discussed in Section 3.1.1 and Section 3.1.2 (i.e., frontal and sagittal mechanisms, respectively). The modified Denavit–Hartenberg convention was applied to obtain the kinematics of the serial linkage portion [57]. Note that the kinematic model of u-Rob was developed on the basis of the anatomy and biomechanics of the human upper limb.

#### 3.1.1. Kinematics of the Frontal Mechanism

To obtain the forward kinematics of the frontal mechanism (please see Figure 3), the following vectors, namely, ${\vec{L}}_{1}$, ${\vec{L}}_{11}$ and ${\vec{L}}_{12}$, were formed, as shown in Figure 3. Using these vectors, the following closed-loop equation (Equation (1)) was formed:

$${\vec{L}}_{1}={\vec{L}}_{11}+{\vec{L}}_{12}$$

(1) $$\left[\begin{array}{c} L_{1}\cos\theta_{1}\\ L_{1}\sin\theta_{1} \end{array}\right]=\left[\begin{array}{c} L_{11}\cos\theta_{11}\\ L_{11}\sin\theta_{11} \end{array}\right]+\left[\begin{array}{c} L_{12}\cos\theta_{12}\\ L_{12}\sin\theta_{12} \end{array}\right]$$

The Equation (1) is a function of $\theta_{1}$ where $L_{11}$, $\theta_{11}$ and $L_{12}$ are known values that depend on the geometry of the function. With these values, the unknowns $L_{1}$ and $\theta_{12}$ can be found.

After rearranging the above equation (Equation (1)), we obtained:

(2) $$\left[\begin{array}{c} L_{1}\cos\theta_{1}\\ L_{1}\sin\theta_{1} \end{array}\right]-\left[\begin{array}{c} L_{11}\cos\theta_{11}\\ L_{11}\sin\theta_{11} \end{array}\right]=\left[\begin{array}{c} L_{12}\cos\theta_{12}\\ L_{12}\sin\theta_{12} \end{array}\right]$$

After squaring both components of the above equation (Equation (2)) and then adding, we obtained the following:

$$\Rightarrow L_{1}^{2}\cos^{2}\theta_{1}+L_{1}^{2}\sin^{2}\theta_{1}+L_{11}^{2}\cos^{2}\theta_{11}+L_{11}^{2}\sin^{2}\theta_{11}-2L_{1}L_{11}\;\cos\theta_{1}\cos\theta_{11}-2L_{1}L_{11}\;\sin\theta_{1}\sin\theta_{11}\;=L_{12}^{2}\cos^{2}\theta_{12}+L_{12}^{2}\sin^{2}\theta_{12}$$

$$\Rightarrow L_{1}^{2}+L_{11}^{2}-2L_{1}L_{11}\left(\cos\theta_{1}\cos\theta_{11}+\sin\theta_{1}\sin\theta_{11}\right)=L_{12}^{2}$$

$$\Rightarrow L_{1}^{2}-2L_{1}L_{11}\;\cos\left(\theta_{1}-\theta_{11}\right)+\left(L_{11}^{2}-L_{12}^{2}\right)=0$$

$$\Rightarrow L_{1}=\frac{2L_{11}\;\cos\left(\theta_{1}-\theta_{11}\right)}{2}\pm \frac{\sqrt{4L_{11}^{2}\;\cos^{2}\left(\theta_{1}-\theta_{11}\right)-4\left(L_{11}^{2}-L_{12}^{2}\right)}}{2}$$

$$\Rightarrow L_{1}=L_{11}\;\cos\left(\theta_{1}-\theta_{11}\right)\pm \sqrt{L_{11}^{2}\;\cos^{2}\left(\theta_{1}-\theta_{11}\right)-\left(L_{11}^{2}-L_{12}^{2}\right)}$$

$$\Rightarrow L_{1}=L_{11}\;\cos\left(\theta_{1}-\theta_{11}\right)\pm \sqrt{L_{11}^{2}\;\cos^{2}\left(\theta_{1}-\theta_{11}\right)-L_{11}^{2}+L_{12}^{2}}$$

(3) $$L_{1}=L_{11}\;\cos\left(\theta_{1}-\theta_{11}\right)+\sqrt{L_{11}^{2}\;\cos^{2}\left(\theta_{1}-\theta_{11}\right)-L_{11}^{2}+L_{12}^{2}}$$

Equation (3) provides the location of the slider, which is the instantaneous center of the shoulder joint.

To obtain the solution for $\theta_{12}$, the sine component of Equation (2) was divided by the cosine component as follows.

$$\tan\theta_{12}=\frac{L_{1}\sin\theta_{1}-L_{11}\sin\theta_{11}}{L_{1}\cos\theta_{1}-L_{11}\cos\theta_{11}}$$

(4) $$\theta_{12}=\arctan\left(\frac{L_{1}\sin\theta_{1}-L_{11}\sin\theta_{11}}{L_{1}\cos\theta_{1}-L_{11}\cos\theta_{11}}\right)$$

Equations (3) and (4) are then used to solve for the forward kinematics of the frontal mechanism.

#### 3.1.2. Kinematics of the Sagittal Mechanism

**Figure 13:**
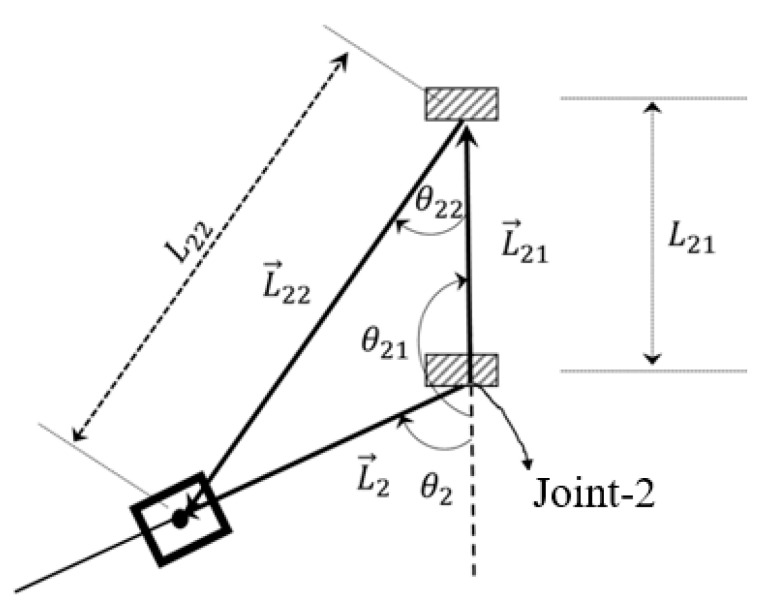
Vector formation of links in the sagittal mechanism.

To obtain the forward kinematics of the sagittal mechanism, the following vectors, namely, ${\vec{L}}_{2}$, ${\vec{L}}_{21}$ and ${\vec{L}}_{22}$ were formed, as shown in Figure 4 and Figure 13. These vectors formed the following closed-loop equation (Equation (5)):

$${\vec{L}}_{22}={\vec{L}}_{2}-{\vec{L}}_{21}$$

(5) $$\left[\begin{array}{c} L_{22}\cos\theta_{12}\\ L_{22}\sin\theta_{12} \end{array}\right]=\left[\begin{array}{c} L_{2}\cos\theta_{2}\\ L_{2}\sin\theta_{2} \end{array}\right]-\left[\begin{array}{c} L_{21}\cos\theta_{21}\\ L_{21}\sin\theta_{21} \end{array}\right]$$

Equation (5) is a function of $\theta_{2}$ where $L_{21}$, $\theta_{21}$ and $L_{22}$ are known values that depend on the geometry of the function. With these values, the unknowns $L_{2}$ and $\theta_{22}$ can be found.

After squaring both components of the above equation (Equation (5)) and then adding, we obtained the following:

$$\Rightarrow L_{22}^{2}\cos^{2}\theta_{22}+L_{22}^{2}\sin^{2}\theta_{22}=L_{2}^{2}\cos^{2}\theta_{2}+L_{2}^{2}\sin^{2}\theta_{2}+L_{21}^{2}\cos^{2}\theta_{21}+L_{21}^{2}\sin^{2}\theta_{21}-2L_{2}L_{21}\;\cos\theta_{2}\cos\theta_{21}-2L_{2}L_{21}\;\sin\theta_{2}\sin\theta_{21}$$

(6) $$L_{2}=L_{21}\;\cos\left(\theta_{2}-\theta_{21}\right)+\sqrt{L_{21}^{2}\;\cos^{2}\left(\theta_{2}-\theta_{21}\right)-L_{21}^{2}+L_{22}^{2}}$$

Equation (6) provides the location of the upper arm attachment.

To obtain the solution for $\theta_{22}$, the sine component of Equation (5) was divided by its cosine component as follows:

$$\tan\theta_{22}=\frac{L_{2}\sin\theta_{2}-L_{21}\sin\theta_{21}}{L_{2}\cos\theta_{2}-L_{21}\cos\theta_{21}}$$

(7) $$\theta_{22}=\arctan\left(\frac{L_{2}\sin\theta_{2}-L_{21}\sin\theta_{21}}{L_{2}\cos\theta_{2}-L_{21}\cos\theta_{21}}\right)$$

Equations (6) and (7) are then used to solve for the forward kinematics of the sagittal mechanism.

#### 3.1.3. Kinematics of the Whole Robot

**Figure 14:**
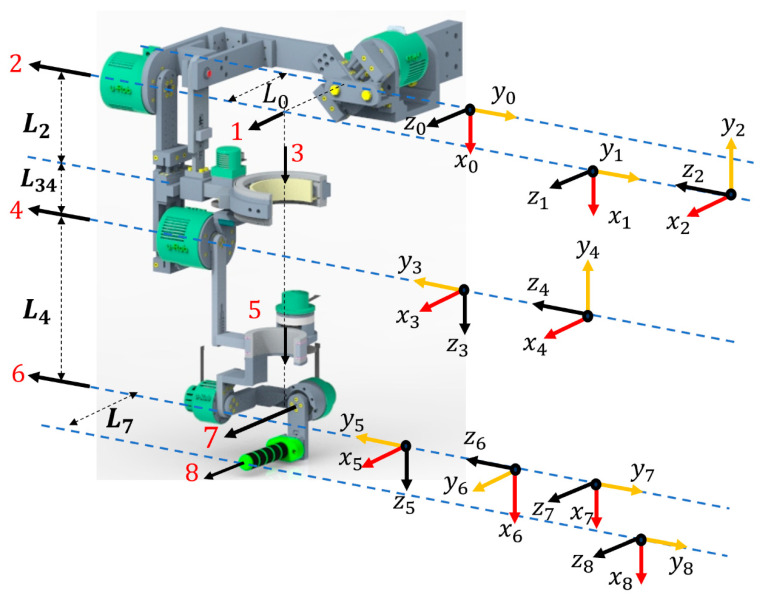
Link frame attachments to the proposed exoskeleton robot.

According to the modified Denavit–Hartenberg (DH) convention [57], the coordinate frames were assigned based on the human arm’s joint axes of rotation. Figure 14 shows the coordinate frame assignment of u-Rob for all of its joints, where black arrowheads indicate the joint axes of rotation of u-Rob corresponding to that of the human upper limb. The modified DH parameters corresponding to the placement of the link frames (as shown in Figure 14) are computed and presented in Table 4.

Using the modified DH parameters, a homogenous transformation matrix between two successive frame {i} and frame {i−1} [57,58] was obtained using the following equation (Equation (8)):

(8) Tii−1=[Rii−13×3Pii−13×101×31]

where Rii−1 is the rotation matrix that describes the frame {i} relative to frame {i−1} and can be expressed as:

(9) Rii−1=[cosqi−sinqi0sinqicosαi−1cosqicosαi−1−sinαi−1sinqisinαi−1cosqisinαi−1cosαi−1]

Furthermore, Pii−1 is the vector that locates the origin of the frame {i} relative to frame {i−1} and can be expressed as:

(10) Pii−1=[ai−1−sαi−1dicαi−1di]

Because of the two parallel mechanisms, the homogenous transformations for frame {1} and frame {2} were obtained using a hybrid approach [59,60]. The transformation for the rest frames can be obtained using the modified DH convention.

Frame {1}:

Using Equations (8)–(10), the following transformation was obtained:

(T10)DH=[cosq1 −sinq100sinq1cosq100001L00001]

However, as mentioned earlier, the slider in the frontal mechanism was initially placed at 45°. This initial placement gives the frame {1} a rotation of 45°, which caused offsets in the x and y positions. Therefore, with the modified DH convention and kinematics of frontal mechanism, the homogenous transformation between frame {1} and frame {0} was obtained as follows:

T10=[cos(q1+π4) −sin(q1+π4)0L1cos(q1+π4) sin(q1+π4)cos(q1+π4)0−L1sin(q1+π4) 001L00001]

Frame {2}:

Using Equations (8)–(10), the following transformation was obtained:

(T21)DH=[cos(q2+π2)−sin(q2+π2)0000−10sin(q2+π2)cos(q2+π2)000001]

Though frame {1} was rotated initially at 45°, frame {2} remained aligned with the upper arm. Therefore, this initial rotation of frame {1} should be adjusted in the homogenous transformation of frame {2} by pre-multiplying the following matrix:

(T21)adjust=[cos(2q1+π4) sin(2q1+π4)00−sin(2q1+π4)cos(2q1+π4)0000100001]

T21=(T21)DH∗(T21)adjust

T21=[cos(2q1+π4) cos(q2+π2)−cos(2q1+π4) sin(2q1+π4)−sin(2q1+π4)0−sin(2q1+π4)cos(q2+π2)sin(2q1+π4)sin(q2+π2)−cos(2q1+π4) 0sin(q2+π2)cos(q2+π2)000001]

The homogenous transformation matrices for the rest frames were found using Equations (8)–(10) as they involved only serial links. 

The homogenous transformation matrix that represents frame {8} with respect to frame {0} was obtained by multiplying individual transformation matrices:

(11) T80=[T10.T21T32.T43.T54.T65.T76T87]=[r11r12r13Pxr21r22r23Pyr31r32r33Pz0001]

The equation obtained from this transformation matrix is known as the forward kinematic equation. With the joint variable of each joint ($q_{1},q_{2},q_{3},q_{4},q_{5},q_{6},\;\mathrm{and}\;q_{7}$), Equation (11) gives the position and orientation of the end-effector frame (wearer’s hand) with respect to the reference (base) frame.

#### 3.1.4. Jacobian

The linear velocity vector of u-Rob’s end-effector frame (i.e., frame {8}) comprises velocities along three Cartesian axes. In contrast, the rotational velocity vector contains angular velocities around three Cartesian axes. From this velocity vector, the Jacobian of u-Rob’s (i.e., *J*(*q*) is a 6 × 7 matrix) end-effector velocities was computed in MATLAB (version R2018a, MathWorks, Natick, MA, USA) with respect to the end-effector frame. In addition, the Jacobian was also calculated with respect to the base frame. Note that u-Rob is a redundant manipulator; hence, its Jacobian is not a square matrix. Using Equation (12), the pseudo-inverse of the Jacobian can be calculated [61]:

(12) $$J_{pseudo-inverse}=J^{T}\left(q\right){\left(J\left(q\right)J^{T}\left(q\right)\right)}^{-1}$$

### 3.2. Dynamics

The dynamic equations of u-Rob were derived from the iterative Newton–Euler formulation as follows [58]:

(13) $$\tau =M\left(q\right)\ddot{q}+V\left(q,\dot{q}\right)\dot{q}+G\left(q\right)+F\left(q,\dot{q}\right)$$

where *M(q)* is the 7×7 mass matrix of the manipulator,$\;V\left(q,\dot{q}\right)$ is a 7×1 dimension vector composed of centrifugal and Coriolis terms, and G(q) is a 7×1 vector of gravity terms. In addition, $F\left(q,\dot{q}\right)$ is a 7 × 1 vector of nonlinear Coulomb friction and can be expressed using the following relation with a coefficient of friction *c*. However, the *M*, *V*, *G*, and *F* matrices are large and hence not included in this work.

(14) $$F\left(q,\dot{q}\right)=c.sgn\left(\dot{q}\right)$$

## 4. Control

To test the desired functionality of the developed exoskeleton robot, a simple PID controller based on state feedback, as shown in Equation (15), was implemented in this research. The trajectory of the exercise was controlled by manipulating the joint torque computed by a PID controller:

(15) $$\tau =K_{P}e+K_{V}\dot{e}+K_{I}\int edt$$

where

$e=q_{d}-q$, $\dot{e}={\dot{q}}_{d}-\dot{q}$,

$K_{P}$, $K_{V}$ and $K_{I}$ are diagonal matrices for the proportional, derivative, and integral gains, respectively.

$q_{d},{\dot{q}}_{d}\in {\mathbb{R}}^{2\times 1}$ are the vectors of the desired joint positions and velocities.

$q,\dot{q}\in {\mathbb{R}}^{2\times 1}$ are the vectors of the actual/measured joint positions and velocities.

The stability of such a control system depends on the proper choice of proportional ($K_{P}$), integral ($K_{I}$), and derivative gains $\left(K_{V}\right)$. The proper choice of these gains makes the controller stable. In this research, the PID gains were set for u-Rob using trial and error. The chosen gains used in the experiment were $K_{P}=$ diag(2200, 1800, 300, 300, 100, 150, 180), $K_{I}=$ diag (50, 40, 30, 25, 18, 15, 15), and $K_{V}=$ diag(20, 18, 16, 15, 10, 8, 7).

**Figure 15:**
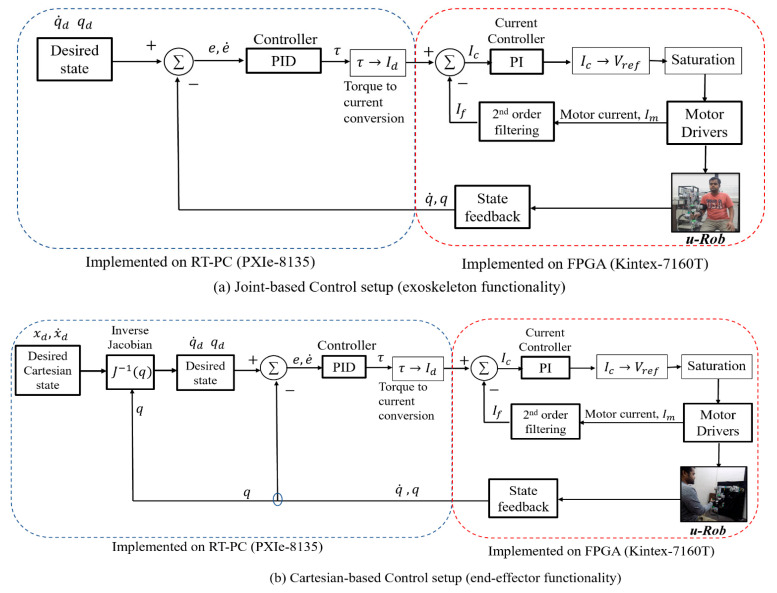
Schematic of control setup of u-Rob for the (**a**) exoskeleton setup and (**b**) end-effector setup.

Joint-based control was implemented for the exoskeleton setup, as shown in Figure 15a, while Cartesian-based control was implemented for the end-effector setup, as shown in Figure 15b. As shown in Figure 15a, for the joint-based control, desired trajectory/states (i.e., joint position and velocity) of the given exercise are sent to the controller. Based on the error calculated from the desired states (i.e., position and velocity) and feedback (i.e., robot joints’ actual position and velocity) obtained from the motor hall sensors, the controller estimates the necessary torques. The torque values are then converted into motor currents using appropriate torque constants. These current values are referred to as the desired currents, which can then be regulated by a PI controller. This controller operates on the error between the desired current and actual/measured current obtained from the current monitors in the motor drivers. The measured current is refined using a second-order filter. The natural frequency and damping ratio used in the filter were 25 rad/s and 0.85, respectively. The estimated currents from the PI current controller are then converted into a reference voltage. Finally, voltage commands are sent to the actuator to maneuver u-Rob’s joints. On the other hand, for the Cartesian-based control, end-effector’s desired position and velocity are converted into desired joint states (position and velocity) using the inverse of the Jacobian and feedback (i.e., robot joints’ actual position and velocity) obtained from the motor hall sensors. Then, the desired joint position and velocities are sent to a controller, which estimates the torque and eventually sends voltage commands to the robot actuators.

**Figure 16:**
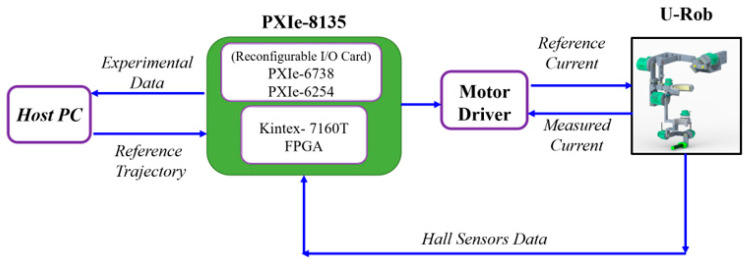
Schematic of the instrumentation for the control of u-Rob.

The instrumentation for the control setup of u-Rob is shown in Figure 16. A host PC and a PXI real-time target are the main elements of the electrical and electronic configuration of the developed u-Rob rehabilitation system. The real-time target consists of a National Instruments PXIe-8135 real-time controller (Industrial PC) with two PXI reconfigurable IO (i.e., PXIe 6738 and PXIe 6254) cards with an embedded FPGA housed in a PXIe-1078 chassis, a mainboard, seven motor driver cards, and actuators. All joint motors are equipped with Hall sensors. The Hall sensors’ data were used to measure the positions of the robot joints. The Hall sensor data was sampled every 100 μs.

A graphical user interface (GUI) on the host PC was developed in LabVIEW (version runtime 2019, National Instruments, Austin, TX, USA). The user can set the developed exoskeleton robot’s home position and initial position and activate and deactivate the joint motor in the GUI. It also lets the operator select the trajectory and type of functionality (i.e., joint-based or Cartesian-based exercise). The input via the user interface in the host PC is sent to the PXIe real-time target, and after the completion of each trajectory run, the data recorded in the PXI real-time target is sent back to the host PC via a file transfer protocol for storage.

## 5. Experiments and Results

**Figure 17:**
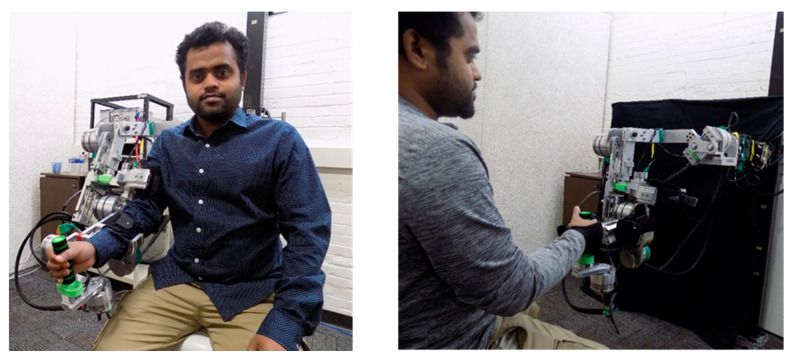
On the **left**, the subject is wearing u-Rob as an exoskeleton-type setup for joint-based exercises, whereas on the **right**, the subject is wearing u-Rob as an end-effector-type setup for end-point exercises.

The functionality of the developed u-Rob exoskeleton system was validated via experiments. Both joint-based exercises and end-point exercises were conducted to validate the exoskeleton-type and end-effector-type functionalities; the experimental setups for both types are shown in Figure 17. The exercises used in the experiments were passive exercises adapted from the recommended library of exercises from the standard rehabilitation therapy protocol [62,63]. The exercises were transformed to a pre-defined trajectory using a cubic polynomial approach for the robot to follow (Craig 2017). The experiment was conducted with five healthy subjects (age: 28 ± 3 years, weight: 165 ± 30 lbs, height: 5 foot 5 inches ± 5 inches). The study was approved by the Institutional Review Board (IRB#:19.064; study title: Experiment of the human natural range of motion with developed robotic device for upper limb rehabilitation). The experiments were conducted for both individual joint and multi-joint movements. To demonstrate the experimental results, plots of the joint position vs. time, error between the reference and actual position, velocity vs. time, and torque vs. time are presented. Moreover, force sensors’ data from one three-axis force sensor instrumented at the wrist joint and three one-axis force sensors instrumented with upper arm cuff are plotted. The red dotted line represents the reference (desired) value for the position and velocity tracking, whereas the solid blue line represents the actual value.

### 5.1. Experimental Results for Joint-Based Exercises (Exoskeleton-Type Setup)

Experiments were conducted for all kinds of exercises with varying speeds. To avoid redundancy, only three individual joint exercises are described in Section 5.1.1, Section 5.1.2 and Section 5.1.3 and two multi-joint exercises are described in Section 5.1.4 and Section 5.1.5. Furthermore, interaction force plots are given only for individual-joint exercises.

#### 5.1.1. Shoulder Abduction–Adduction Exercise

**Figure 18:**
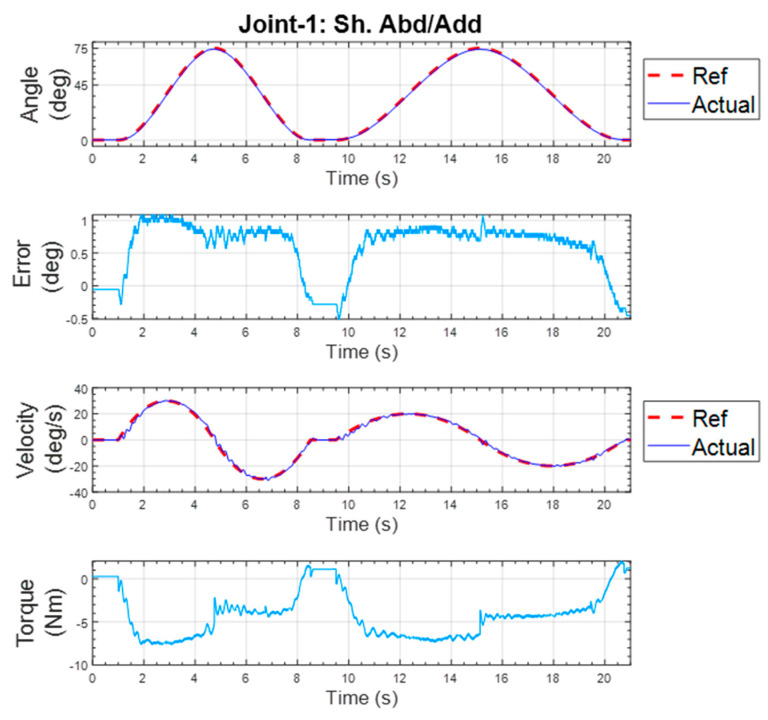
Individual joint exercise for shoulder abduction–adduction.

This repetitive exercise was initiated with all joints at the zero position, and then the shoulder was abducted to 75° and returned to 0°. After a second, the same movement was repeated with a slower velocity. The result of the experiment is shown in Figure 18. From the figure’s topmost plot, it is clearly seen that the actual position and reference position almost overlapped, meaning the proposed exoskeleton robot followed the given (reference) position. The maximum error for the position tracking was found to be 1.09°, which shows the excellent tracking performance of the controller. The maximum velocities during the first and second repetitions were 30 deg/s and 20 deg/s, respectively.

**Figure 19:**
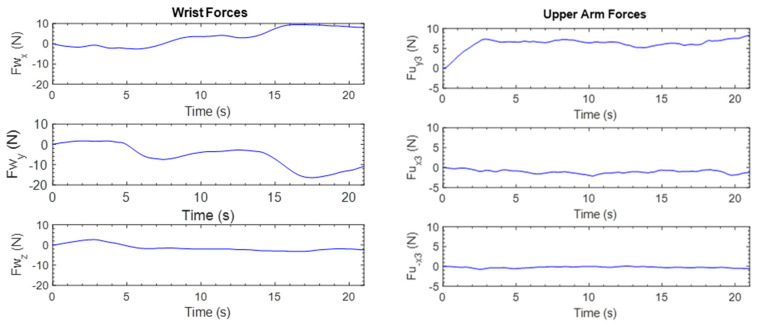
Subject’s forces during shoulder abduction and adduction.

The forces exerted by the subject at the wrist and upper arm are shown in Figure 19. The figure shows that the subject interacted mostly at the *x*- and *y*-axes of the end-effector. At the upper arm, most interactions happened in the positive y3-direction of the end-effector. These results demonstrate the interaction/resistance between the subject and the proposed exoskeleton robot. This resistance can be quantified and read on an appropriate scale to measure the user’s discomfort, arm stiffness, and so on.

#### 5.1.2. Shoulder Vertical Flexion–Extension Exercise

**Figure 20:**
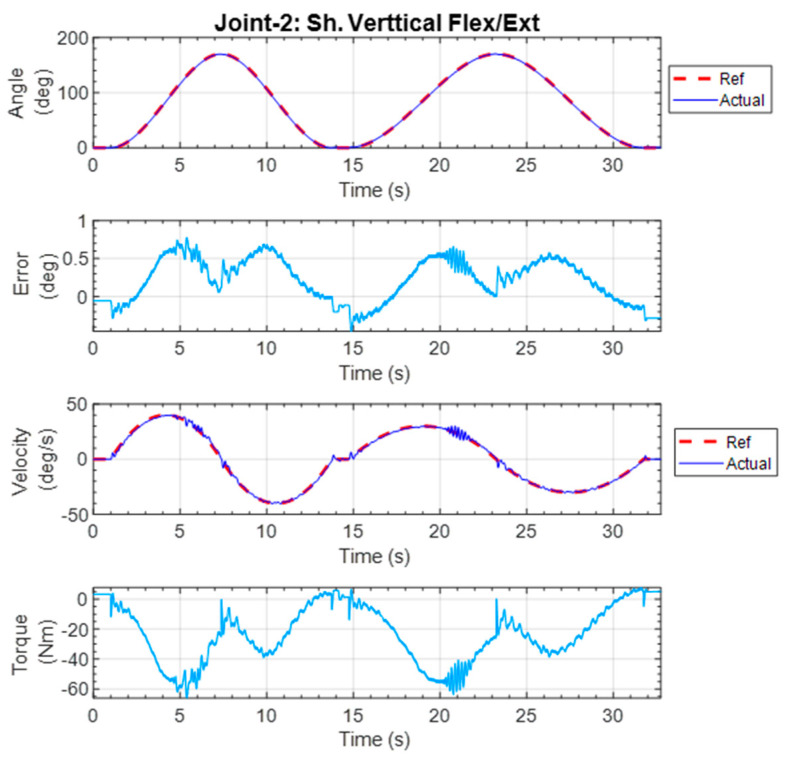
Individual joint exercise for shoulder vertical flexion–extension.

**Figure 21:**
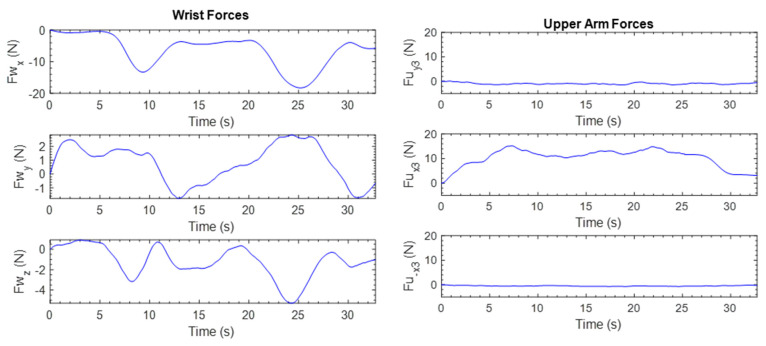
Subject’s forces during shoulder vertical flexion–extension.

This repetitive exercise was initiated with all joints at the zero position, and then the shoulder was vertically flexed to 170° and returned to 0°. The exercise was repeated with a slower velocity. The experimental results are shown in Figure 20. The maximum error for the position tracking was found to be around 0.91°, which shows the excellent tracking performance of the controller. The maximum velocities during the first and second repetitions were 60 deg/s and 45 deg/s, respectively. The force plots are shown in Figure 21. It is seen that the subject mostly exerted forces along the x3-axis at the upper arm. The force sensor interacted with the subject the most during the shoulder joint vertical flexion.

#### 5.1.3. Wrist Flexion–Extension Exercise

**Figure 22:**
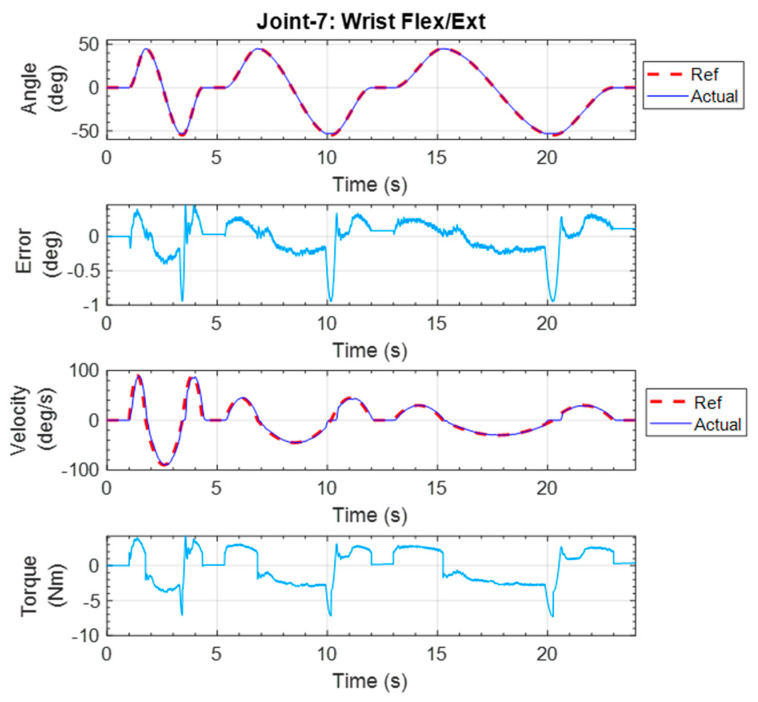
Individual joint exercise for wrist flexion–extension.

This repetitive exercise was initiated with the elbow joint angle at a 90° angle and maintained that position during the experiment. All other joints remained at the zero position. From the initial position, the wrist was extended to 55° and then flexed to 50°. The exercise ended with the wrist returned to the initial position. The trajectory tracking results are shown in Figure 22. Once again, the error of the position tracking was observed to be less than 1°.

#### 5.1.4. Simultaneous Joint Movement of the Shoulder, Elbow, and Wrist

**Figure 23:**
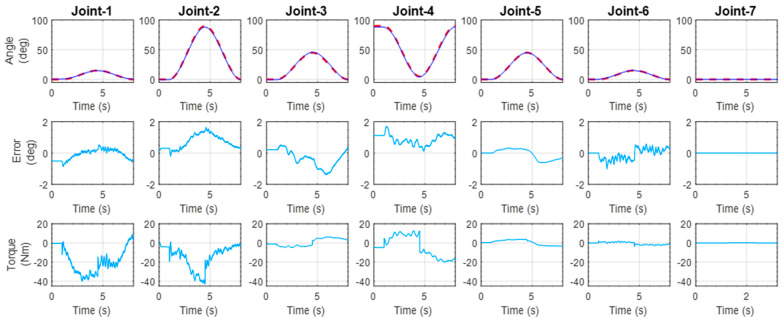
Experimental result for the movement (a diagonal reaching) of all joints, except joint-7.

This exercise involved the simultaneous movement of all joints, except joint-7 (wrist flexion–extension). It replicated a diagonal reaching movement that started moving from an initial position (all joints were at 0° while the elbow was at a 90° position) to the reaching position (abduction 15°, vertical flexion 90°, external rotation 45°, elbow flexion 10°, forearm pronation 45°, and wrist ulnar deviation 15°), and then returned to the initial position. As observed from Figure 23, the results show that the developed exoskeleton robot followed the reference trajectory. From the figure, it is seen that the position error for all the joints remained below 2°. The maximum error (1.85°) was found for the elbow joint.

#### 5.1.5. Diagonal Reaching Exercise

**Figure 24:**
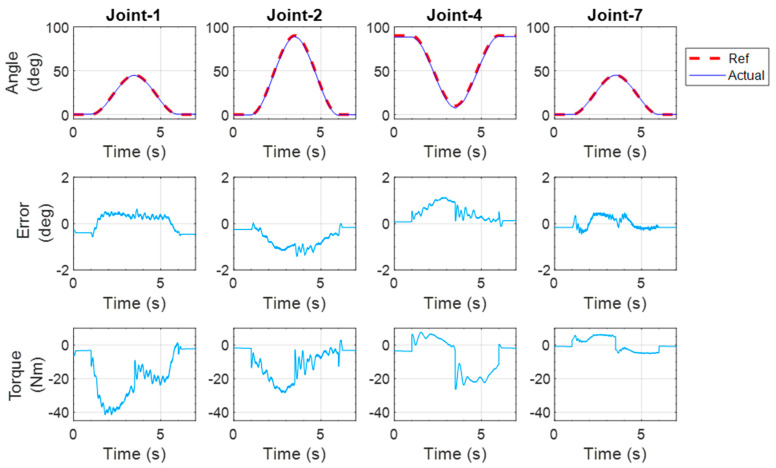
Experimental results of the diagonal reaching exercise.

This diagonal reaching exercise comprised shoulder, elbow, and wrist joints movements. The exercise was initiated with the elbow at 90°. Then, the shoulder joint was abducted from 0° to 45° and adducted back to 0° at the end of the exercise. The shoulder was also vertically flexed from 0° to 90° and extended back to 0° at the end of the training. During the movement of the shoulder, the elbow was extended to 90° and returned to 0°. In the meantime, the wrist was extended to 50° and returned to zero. The experimental results are shown in Figure 24. The maximum error between the reference and actual position was observed to be around 1.81° for joint 4.

### 5.2. Experimental Results for End-Point Exercises (End-Effector-Type Setup)

To conduct end-point exercises with the developed u-Rob, Cartesian control was used. In Cartesian control, the proposed exoskeleton robot was given the positions and orientation of the end-effector. Using a cubic polynomial, these positions and orientations were then transformed into end-effector Cartesian velocities. The inverse kinematic solution was obtained for u-Rob with these velocities using an inverse Jacobian. The control architecture of the Cartesian control is depicted in Figure 15. In the following subsections, the experimental results of three reaching exercises are presented to show the end-effector-type functionality of the developed u-Rob.

#### 5.2.1. Reaching Exercise in the Transverse Plane

**Figure 25:**
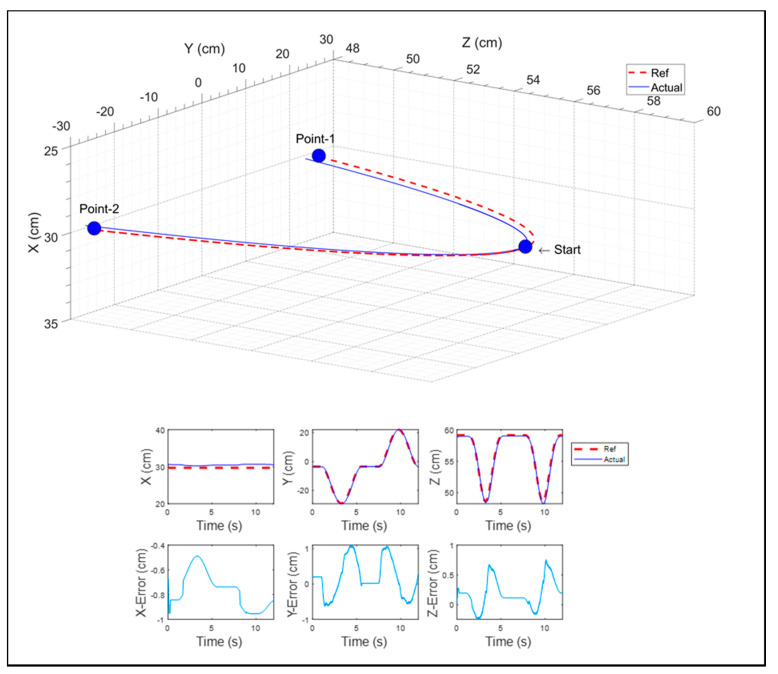
Reaching in the transverse plane.

In this exercise, while carrying the subject’s limb, the exoskeleton robot moved from a point to another point in the transverse plane, as shown in Figure 25. The top plot shows the position tracking of the end-effector in 3D space, whereas the bottom plots show the Cartesian trajectory tracking of the exoskeleton in terms of the x, y, and z positions and the corresponding tracking errors. This kind of motion resembled tasks like wiping a table. The end-effector position was tracked nicely; the maximum error found was 1 cm, which occurred in the *y*-axis.

#### 5.2.2. Forward Reaching in the Sagittal Plane

**Figure 26:**
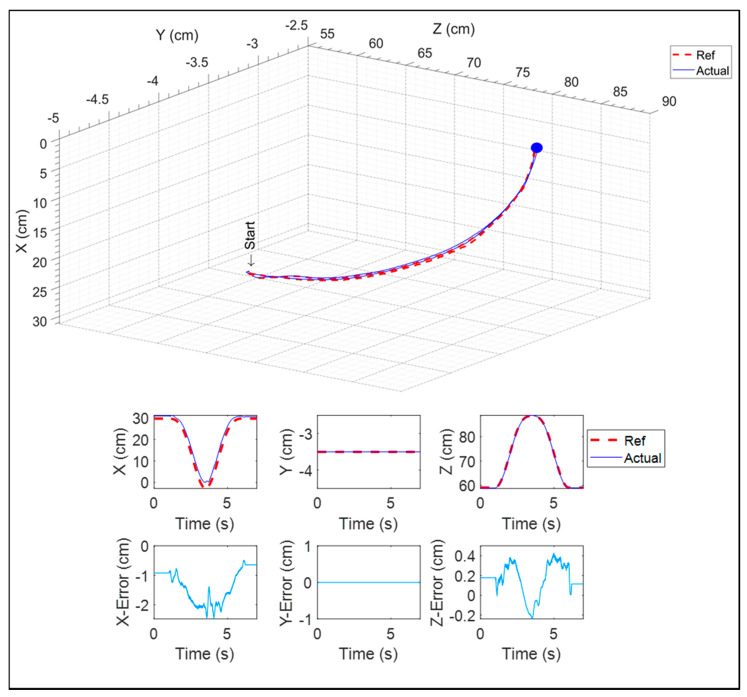
Forward reaching in the sagittal plane.

This kind of exercise is similar to pulling or pushing an object (e.g., opening a door). The end-effector reached the target (blue marker) and then returned to the initial position. The experimental results for this exercise are shown in Figure 26. The maximum error (2.12 cm) was found in the x8-direction.

#### 5.2.3. A 3D Reaching Exercise

**Figure 27:**
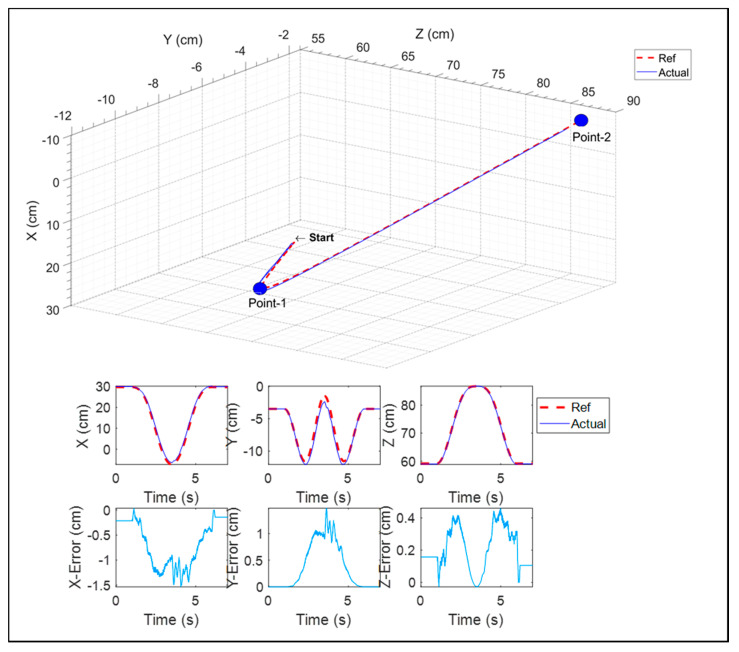
A 3D reaching task.

In this exercise, as shown in Figure 27, the end-effector reached a point (i.e., point-1) in 3D space from the start position. After that, it went to point-2. The result shows excellent tracking with an error below 1.5 cm.

Looking at the results from all the experiments, it was concluded that the developed u-Rob robot could function as both an exoskeleton-type and end-effector-type robot. Furthermore, experimental results show that the PID controller could efficiently run u-Rob with negligible tracking error to perform a variety of rehabilitation exercises involving single-joint movement, multi-joint movements, and Cartesian-based movement. Thus, u-Rob should be adequate for performing passive rehabilitation of an impaired upper limb.

## 6. Conclusions

This research developed a seven-DoF upper limb rehabilitation robot that features dual functionality, a novel sensorized upper arm cuff, and an ergonomic shoulder with a movable center of rotation. A hybrid approach was used to obtain the kinematic equations of the developed u-Rob. Safety was confirmed by designing the neighboring links to act as mechanical stoppers and setting thresholds for the ROMs, velocities, and interaction forces and torques. PID control algorithms were implemented to control the developed robot. From the experimental results, it was observed that the tracking error remained significantly low for both joint-based exercises and end-point exercises to validate the exoskeleton-type and end-effector-type functionalities of the developed u-Rob. Potential future works include developing a controller and experimenting with active rehabilitation exercises.

## Figures and Tables

**Table 1:** Comparison of the ranges of motion of the proposed exoskeleton robot with existing robots.

Limb Segment	Joint No.	Kind of Motion	ROM
Shoulder	Joint-1	Abduction	90°
		Adduction	0°
	Joint-2	Vertical flexion	180°
		Vertical extension	0°
	Joint-3	Internal rotation	90°
		External rotation	90°
Elbow and Forearm	Joint-4	Flexion	135°
		Extension	0°
	Joint-5	Pronation	90°
		Supination	90°
Wrist	Joint-6	Flexion	60°
		Extension	50°
	Joint-7	Radial deviation	20°
		Ulnar deviation	30°

**Table 2:** Design specifications and selected components of the developed u-Rob.

**Degrees of Freedom**						
Active				Passive		
7				2		
Ranges of motion/joints’ limits (degrees)						
Joint-1	Joint-2	Joint-3	Joint-4	Joint-5	Joint-6	Joint-7
0 to 90	0 to 180	−90 to 90	0 to 135	−90 to 90	−60 to 50	−20 to 30
**Fabrication**						
Material		Aluminum 6061, stainless steel 304, plastic (polylactic acid and polycarbonate)				
Fabrication process		CNC machining, lathe turning, 3D printing				
**Actuators**						
Location		Joint-1, 2, 4		Joint-3	Joint-5, 6, 7	
Motors		Maxon EC90, 90 W		Maxon EC45, 70 W	Maxon EC45, 45 W	
Operating voltage (V)		24		24	24	
Nominal speed (rpm)		2590		4860	2940	
Nominal current (A)		6.06		3.21	1.01	
Torque constant (mNm/A)		70.5		36.9	51	
Nominal torque (mNm)		444		128	55.8	
Weight (g)		600		147	75	
Motor drivers		ZB12A8 analog servo drive				
Motor driver current rating (A)		12 (peak) 6 (continuous)				
Motor driver input		Analog (voltage)				
Motor driver feedback		Current sense, Hall sensor pulses				
**Reducers**						
Location		Joint-1, 2, 4		Joint-3	Joint-5, 6, 7	
Gear reducer		Harmonic drive CSF-17-100-2UH		Harmonic drive CSF-11-100-2XH-F	Leader drive LHSG-14-C-I	
Reduction ratio		100		100	100	
Average output torque (Nm)		39		8.9	13.5	
Momentary peak torque (Nm)		108		25	66	
Repeated peak torque (Nm)		54		11	34	
Estimated max output speed (deg/s)		210		290	155	
**Control System**						
Controller		NI PXIe-8135				
Data acquisition cards		Two PXIe-6738, 6254 reconfigurable IO cards				
Control architecture		Ni RT Linux real-time CPU execution + FPGA				
CPU		Intel Atom 1.6 GHz quad-core				
Memory		4 GB				
FPGA		Kintex-7 70T FPGA				
Input/output		5 V TTL digital logic I/O, ±10 V analog in/out				
Communication		Ethernet, EtherCAT, CANopen, RS485, RS232				
**Force sensors**						
Location		End effector		Upper arm cuff		
Sensor		GPB160-50N, GALOCE		TAS606, HT Sensor Technology		
Sensor type		3-axis load cell		Single-axis load cell		
Measuring capacity		Fx, Fy, Fz = 50 N		50 N		

**Table 3:** Mass inertia properties of the proposed exoskeleton system.

Segment	Segment Length (mm)	Segment Weight (kg)	Center of Gravity *CG* (mm)			Moment of Inertia *I at CG* (kg·mm^2^) (10^3^)		
			*CG_X_*	*CG_Y_*	*CG_Z_*	*Ixx*	*Iyy*	*Izz*
Segment-1 (joint-1 to joint-2)	231.4	4.93	−6.65	−221.5	−63.6	118.5	31.5	94.4
Segment-2 (joint-2 to joint-3)	183.5 ± 50	1.12	−8.95	−10.95	17.3	47.2	25.7	24.3
Segment-3 (joint-3 to joint-4)	82.04	3.35	−10.9	13.87	−27.7	40.06	14.09	32.94
Segment-4 (joint-4 to joint-5)	163.5 ± 40	1.24	−57.6	−142.3	40.6	4.27	4.64	3.74
Segment-5 (joint-5 to joint-6)	132.775	1.34	−18.2	83.2	−48.6	9.45	5.12	7.68
Segment-6 (joint-6 to joint-7)	92.76	1.08	−0.55	−92.26	33.8	4.54	2.93	2.24
Segment-7 (joint-7 to wrist handle)	47	0.22	23.8	0.00	−80.9	0.00683	0.036	0.037

**Table 4:** Modified Denavit–Hartenberg parameters for the proposed exoskeleton robot.

Joint *(i)*	*α_i−_*_1_ *(Link Twist)*	*d_i_* *(Link Offset)*	*a_i−_*_1_ *(Link Length)*	*q_i_* *(Joint Variable)*
1	0	0	*L*_0_	*q*_1_
2	*π*/2	0	0	*q*_2_*+π*/2
3	*π*/2	*L*_2_ * + L*_34_	0	*q*_3_
4	*−π*/2	0	0	*q*_4_
5	*π*/2	*L*_4_	0	*q*_5_
6	*−π*/2	0	0	*q*_6_*−π*/2
7	*−π*/2	0	0	*q*_7_
8	0	0	*L*_7_	0
